# Supplementary material for: MetaRibo-Seq measures translation in microbiomes
Source: Nat Commun. 2020 Jun 29;11:3268. doi: 10.1038/s41467-020-17081-z (PMC7324362; doi:10.1038/s41467-020-17081-z)
Supplement: Supplementary file 10 — Supplementary Data 7 [file 41467_2020_17081_MOESM10_ESM.zip › File2/Confidence_High_Taxonomy/84791_out.krona.html]

Javascript must be enabled to view this page.

members
magnitude
magnitudeUnassigned
count
unassigned
taxon
rank

84791\_out

5

2759
superkingdom
2

4751
kingdom
2

2
451864
subkingdom

4890
phylum
2

147537
subphylum
2

class
4891
2

4892
order
2

2
115784
family

2
genus
83173

2
56415
species

SRS013687\_contig\_number\_19790SRS1041145\_contig\_number\_15505


SRS023914\_contig\_number\_contig-100\_1935.158396SRS051031\_contig\_number\_contig-100\_1099.195958SRS098571\_contig\_number\_46932
3
